# Identification of symptom clusters and change trajectories in patients with acute exacerbation of chronic obstructive pulmonary disease

**DOI:** 10.1016/j.heliyon.2024.e33745

**Published:** 2024-07-04

**Authors:** Mengying Xu, Chunchun Yu, Xiaona Lin, Jiaqi Shi, Xinyue Pang, Zhi Chen, Hongjun Zhao, Chengshui Chen

**Affiliations:** a Key Laboratory of Interventional Pulmonology of Zhejiang Province, Department of Pulmonary and Critical Care Medicine, The First Affiliated Hospital of Wenzhou Medical University, Wenzhou, 325000, China; b Zhejiang Province Engineering Research Center for Endoscope Instruments and Technology Development, Clinical Research Centre, Department of Pulmonary and Critical Care Medicine, Quzhou People's Hospital, The Quzhou Affiliated Hospital of Wenzhou Medical University, Quzhou, 324000, China

**Keywords:** Acute exacerbation of chronic obstructive pulmonary disease, Symptom clusters, Nursing, Physiological mechanism

## Abstract

**Aim and objectives:**

This study aimed to identify symptom cluster (SC) patterns and change trajectories in patients with acute exacerbation of chronic obstructive pulmonary disease (AECOPD), the correlation of the SCs with laboratory and imaging indicators, and the intrinsic association of the SCs with prognostic outcomes and disease burden.

**Method:**

Symptom information was collected using a digital evaluation scoring system at the time of admission, on the third day after admission, and upon discharge. Laboratory and imaging examination data were compiled simultaneously. Exploratory factor analysis was used to identify the AECOPD SCs. The number of factors (clusters) was determined by examining factors with eigenvalues ≥1.0, using 0.50 for factor loadings as the minimum cut-off value. Spearman's correlation analysis was used to explore the link between the SCs and laboratory and imaging indicators, as well as the relationship between the severity of the symptoms in different clusters, prognostic outcomes, and disease burden.

**Results:**

This study included 148 patients. Three SCs were identified: activity-nutrition SC, breath-sleep SC and respiratory SC. Correlation analysis indicated a connection between the activity-nutrition SC and the white blood cell count, and serum sodium and potassium levels, whereas the breath-sleep SC was correlated with white blood cells and eosinophil counts, serum potassium level, and pleural effusion. Additionally, the respiratory SC was associated with serum calcium and magnesium levels, the partial pressure of carbon dioxide, and C-reactive protein (CRP) level. There was a positive correlation between the activity-nutrition SC and hospitalization cost, as well as between the breath-sleep SC and both the hospitalization length and cost.

**Conclusion:**

Patients with AECOPD presented three SCs that affected the length and cost of hospitalization. Concurrently, the severity of the symptoms in the clusters was related to white blood cell and eosinophil counts; serum sodium, potassium, calcium, and magnesium levels; CRP level; the partial pressure of carbon dioxide; and pleural effusion, indicating that the symptoms in each clusters may share related physiological mechanisms. An in-depth exploration of the pathogenesis and intervention paths of health problems is of great significance for promoting precision nursing.

**What is know?**

● Patients with acute exacerbation of chronic obstructive pulmonary disease (AECOPD) experience a variety of symptoms. These symptoms may be interconnected and constitute the ‘symptom cluster’ (SC), which affects patients' quality of life and disease burden.
● It is vital for nurses to identify the interrelatedness and change in symptoms to guide timely nursing.

**What is new?**

● AECOPD-related symptoms are temporally dynamic. The identification of the AECOPD SCs and their trajectories helps to formulate SC-based interventions and implement precision nursing.
● This study provides new insights into the underlying mechanisms of SCs.

## 1 Introduction

Chronic obstructive pulmonary disease (COPD) refers to a variety of lung conditions caused by airway abnormalities (bronchitis and bronchiolitis), alveoli (emphysema), or both. These abnormalities result in persistent and often progressive airflow obstruction, manifesting in symptoms of chronic respiratory distress including coughing, expectoration, and/or exacerbations [1]. In recent years, COPD has emerged as a significant public health issue worldwide due to its high mortality, disability, and prevalence rates [2]. Its prevalence in the Chinese population has reached 8.6 % among individuals aged 20 years, 13.7 % among those aged 40 years and above, and more than 27.0 % among those aged 60 years and above [3]. The mortality rate associated with COPD in the Chinese population is higher than the global average owing to factors such as increased cigarette smoke exposure, air pollution, and an aging population [4]. In 2017, 3.2 million people died from COPD, with the projected annual deaths expected to reach 4.4 million by 2040, indicated a growing burden of COPD in the future [5]. Because COPD is incurable, the objectives of treatment are to manage symptoms, reduce exacerbation and improve the quality of life [6].

Acute exacerbation of chronic obstructive pulmonary disease (AECOPD) is a pivotal event in the course of COPD that adversely impacts disease progression, comorbidities and overall health. An average of 0.5–3.5 acute exacerbations occur annually in each patient with COPD [7]. Patients with COPD often die from acute exacerbations, which incur significant medical costs [8]. After contracting a bacterial or viral infection, patients with AECOPD report increased symptoms 2–3 days later [9], which last for 7–14 days or longer. Following recovery, some patients may not return to baseline condition and instead enter a cycle of frequent exacerbations [10]. Therefore, it is critical to develop efficient strategies to reduce the burden of symptoms for patients with AECOPD. To improve clinical symptom management and reduce the risk of acute exacerbations and readmissions, symptom management science advocates promotes a transition from addressing single symptom to emphasizing symptom clusters (SCs) as a vital part of the development of clinical nursing practice [11].

## 2 Background

Patients with COPD frequently experience a range of upsetting symptoms [12], which rarely occur in isolation. A cross-sectional study found that the average number of symptoms experienced by patients with COPD with moderate to severe airflow obstruction was 7.9 ± 4.3, and this high number of symptoms significantly impacted their quality of life [13]. AECOPD is the acute progressive stage of COPD, defined as “an event characterized by worsening dyspnea and/or cough and sputum over 14 days, which may be accompanied by tachypnea and/or tachycardia, and is often associated with increased local and systemic inflammation caused by airway infections, pollution, or other insult to the airways" [1]. Individuals with COPD typically seek nursing care or treatment upon the exacerbation of their symptoms. Meanwhile, Patients with AECOPD experience more complex and heterogeneous symptoms [14], and multiple symptoms may synergise and strengthen each other to constitute SCs throughout the disease progression, which can have a cumulative or multiplicative effect on health outcomes [15]. An interdisciplinary working group led by Miaskowski et al. [16], emphasized the importance of considering the patient's symptom experience, the time-related characteristics of symptoms within a cluster, and the phenotypic and molecular mechanisms associated with symptoms when defining symptom clusters. This implies that it is necessary to investigate the stability of symptoms within a cluster at various stages and their correlation with pathogenic mechanisms. Furthermore, recent evidence suggests that diverse symptoms can be relieved using the same intervention strategy. For instance, exhaustion and dyspnea are treated using pulmonary rehabilitation [17]. Consequently, identification of SCs has become a prominent and developing area of scientific research.

To achieve the optimal assessment and management of patients with COPD, researchers are urged to identify SCs associated with this disease [11]. Nevertheless, it is challenging to investigate the diversity of symptom-related interactions due to the limited research on SCs in patients with AECOPD exist and most of them are cross-sectional studies. To provide nurses with a foundation for enhanced symptom management, this study aimed to identify the SCs and their change trajectories in patients with AECOPD, investigate the relationship between SCs and imaging and laboratory indicators, as wall as to determine the relationship between SCs and prognostic outcome and disease burden.

## 3 Method

### 3.1 Sampling and design

This longitudinal observational study was conducted at a tertiary hospital in Wenzhou, following the guidelines of Strengthening the Reporting of Observational Studies in Epidemiology (STROBE). Patients with AECOPD admitted through emergency visits or self-admission to the respiratory department or respiratory intensive care unit between July 2022 and August 2023 were selected using convenience sampling. The inclusion criteria were as follows [18]: (1) a definitive AECOPD diagnosis according to the Chinese Expert consensus on the Diagnosis and Treatment of Acute Exacerbation of Chronic Obstructive Pulmonary Disease (2017 Update): necessitates a change in the patient's medication regimen due to symptoms such as coughing, shortness of breath, and abnormal sputum production; (2) Age ≥18 years old; and (3) Clear consciousness and normal communication skills. We excluded the following patients: (1) patients with other lung diseases such as pulmonary embolism, bronchial asthma, bronchiectasis or lung cancer; (2) patients with worsening symptoms due to other diseases such as heart failure; (3) patients with mental illness or cognitive impairment; and (4) patients with major organic diseases.

The sample size for this study was determined using the formula：*n = Z*^*2*^_*α/2*_*(1-P)P/δ*^*2*^. The acceptable error range (‘*δ*’) was set at 0.05, whereas the test level (‘*α*’) was set to 0.05 (*Z*_*α/2*_ = 1.96) [19]. The highest incidence of symptoms among patients with AECOPD was 90 % (*P* = 0.90), as per the early results. Thus, for this study, a minimum sample size of 1.962 × (1–0.90) × 0.90 ÷ 0.052 = 70 was needed. In total, 157 patients were enrolled in this study. A total of 148 patients remained after removing 1 newly diagnosed case of bronchiectasis, and 8 newly diagnosed cases of lung cancer. This clinical study was approved by the Medical Ethics Committee of the hospital (KY2023-R084), and all patients provided informed consent.

### 3.2 Measurement instruments

#### 3.2.1 General information questionnaire

The general information questionnaire created by the investigator was used to collect data on demographic (name, sex, age, current residence, current occupation, educational level, and marital status) and disease (smoking history, history of frequent exacerbations, pathogenic occupational environment, COPD course, body mass index, and infection) characteristics.

#### 3.2.2 Digital evaluation score system (DESS)

The DESS is a semi-quantitative scoring system developed by Professor Wang et al. [20], with the main purpose of digitizing textual data for optimizing the utilization of patient information. The content comprises three main sections: symptom assessment, laboratory and imaging examinations. Each variable was scored on a scale of 0–4. Descriptive clinical information, such as ‘none/little/moderate/a lot’, or ‘none/yes’, can be replaced by ‘0/1/2/4’ or ‘0/4’. A normal physiological range is indicated by a minimum value of 0, whereas a maximum value of 4 indicates a situation far above or worse than the normal range. Each variable was scored using established scoring systems, including the COPD assessment test, acute physiology and chronic health evaluation, BODE index, and diagnostics. Abandoned the 3 points since the score multiples growth can more accurately indicate the severity of the symptoms. Applying the DESS to SCs studies has the benefit of providing more comprehensive information that can be used to investigate clinical biomarkers and influencing factors. In addition, the scale was more effectively and clinically implemented.

### 3.3 Procedures

Separate datasets were collected on the day of admission (T1), the third day after admission (T2), and at discharge (T3).

First evaluation (T1): Demographic information and disease characteristics were collected within 24 h of admission. DESS was used to evaluate the patients' symptoms, and preliminary biological and imaging data were obtained.

Second evaluation (T2): Considering the average length of stay of patients with AECOPD [21], to better reflect the existence and intensity of symptoms, the severity of symptoms was re-examined using the DESS on the third day after admission.

Third evaluation (T3): Patients were assessed using the DESS on the day of hospital discharge following improvement. Data on the length and cost of hospitalization were collected simultaneously. Ultimately, the end event of the follow-up was readmission for COPD exacerbations, and the data for this event were gathered 3 months after discharge using either telephone follow-up or access to the electronic medical record system.

The questionnaires were immediately returned to the researchers for safekeeping once completed by the patients. All the data were reviewed and sorted out by the researchers.

### 3.4 Statistical analysis

All statistical analyses were conducted using IBM SPSS version 25.0. Exploratory Factor Analysis (EFA) was used to identify AECOPD SCs. After confirming the factorability of the data through the Kaiser-Myer-Olkin (KMO) test and the significance of Bartlett's test of sphericity, varimax rotation was used to extract the components from a principal component analysis for the EFA [22]. The number of factors (clusters) was ascertained by examining the factors with eigenvalues ≥1.0, using 0.50 for factor loadings as the minimum cut-off value [23]. The relationship between the SCs and the imaging and laboratory indicators, as well as the relationship between the SCs, prognostic outcomes, and disease load, were examined using Spearman's correlation analysis. All results were two-tailed, with a significance level of P < 0.05 deemed statistically significant.

## 4 Results

### 4.1 Participant characteristics

The average age of the patients in this study was 73.71 years (standard deviation = 9.39). Twenty-one patients (14.19 %) were female, and 127 (85.81 %) were male.

Regarding disease characteristics, 71.62 % of the patients had a history of smoking, and 36 were smokers. Of these patients, 43.34 % had been diagnosed with COPD for more than 10 years. Thirty-nine patients had experienced acute exacerbations at least two times in the past year. Of these, 22.98 % had bacterial, viral, fungal, or mixed infections, as illustrated in Table 1.

**Table 1:** Demographic and disease characteristics of the participants.

Characteristics	classification	n	%
**Sex, n(%)**	Male	127	85.81
	Female	21	14.19
**Age in years, n(%)**	40–59	10	6.76
	60–69	41	27.70
	70–79	52	35.14
	≥80	45	30.40
Maritial **status**, **n(%)**	Married	141	95.27
	Divorced	2	1.35
	Widowed	5	3.38
**Educational level, n(%)**	≤Elementary school	117	79.05
	Middle school	18	12.16
	High school/secondary school	11	7.43
	≥College	2	1.36
**Current residence, n(%)**	Village	93	62.84
	Towns	55	37.16
**Current occupation, n(%)**	Worker	14	9.46
	Farmer	28	18.92
	Retired	41	27.70
	Unemployed	63	42.57
	Other	2	1.35
**Body Mass Index(kg/m**^**2**^**), n(%)**	＜18.5	42	28.38
	18.5–23.9	87	58.78
	＞23.9	19	12.84
**Smoking history, n(%)**	Never smoker	42	28.38
	Former smoker	70	47.30
	Current smoker	36	24.32
**Time since diagnosed (Years), n(%)**	＜5	61	41.22
	5∼10	23	15.54
	＞10	64	43.34
**Pathogenic occupational environment, n(%)**	Yes	28	18.91
	No	120	81.09
**Frequent exacerbations, n(%)**	Yes	39	26.35
	No	109	73.65
**infection, n(%)**	No	114	77.02
	Bacteria	10	6.76
	Fungus	6	4.05
	Virus	15	10.14
	Mixed infection	3	2.03

[note]:Frequent exacerbations were defined as two or more acute exacerbations within the previous year.

### 4.2 Prevalence and severity of symptoms

Twenty symptoms were reported in patients with AECOPD upon admission, with prevalence ranging from 2.70 % to 97.30 %. The most commonly occurring symptom was limitation of activity (97.30 %), followed by shortness of breath (93.92 %), cough (91.22 %), decreased exercise tolerance (89.86 %), chest distress (89.19 %), and sputum (89.19 %), as shown in Table 2.

**Table 2:** Prevalence and severity of symptoms at admission.

Symptoms	Prevalence,n (%)	Severity (median, interquartile)
**Limitation of activity**	144 (97.30)	2.00 (2.00,4.00)
**Shortness of breath**	139 (93.92)	2.00 (2.00,2.50)
**Cough**	135 (91.22)	1.00 (1.00,2.00)
**Decreased exercise tolerance**	133 (89.86)	4.00 (4.00,4.00)
**Chest distress**	132 (89.19)	2.00 (2.00,2.00)
**Sputum**	132 (89.19)	2.00 (1.00,4.00)
**Fatigue**	117 (79.05)	4.00 (4.00,4.00)
**Loss of appetite**	105 (70.95)	1.00 (0.00,2.00)
**Insomnia**	77 (52.03)	4.00 (0.00,4.00)
**Weight loss**	70 (47.30)	0.00 (0.00,1.00)
**Cannot lay down at night**	59 (39.86)	0.00 (0.00,4.00)
**Constipation**	47 (31.76)	0.00 (0.00,4.00)
**Anxiety**	44 (29.73)	0.00 (0.00,1.00)
**Pain**	29 (19.59)	0.00 (0.00,0.00)
**Fever**	28 (18.92)	0.00 (0.00,0.00)
**Hoarse**	23 (15.54)	0.00 (0.00,0.00)
**Limb swelling**	22 (14.86)	0.00 (0.00,0.00)
**Dysphoria**	16 (10.81)	0.00 (0.00,0.00)
**Drowsiness**	13 (8.78)	0.00 (0.00,0.00)
**Dysphagia**	4 (2.70)	0.00 (0.00,0.00)

### 4.3 Prevalence and severity of abnormal laboratory and imaging indicators

Emphysema (85.14 %), neutrophil count (81.76 %), tumor markers (75.00 %), serum calcium level (74.32 %), and the partial pressure of carbon dioxide (62.84 %) were the five indicators with the highest prevalence among the aberrant laboratory and imaging findings in patients, as shown in Supplementary Table 1.

### 4.4 Symptom clusters in patients with AECOPD

Symptoms with a frequency of ≥40 % were chosen for the EFA based on literature review [[24], [25], [26], [27]]. A KMO of 0.73, and a significant results from Bartlett's test of sphericity (*P* < 0.001) indicated that the correlation matrix was suitable for subsequent factor analysis. Finally, three factors were extracted based on the principle of factor eigenvalue ≥1.00 and symptom load ≥0.50 under each factor. The variance contributions were 33.47 %, 13.29 %, and 10.63 %, with a combined total contribution rate of 57.39 %. As shown in Table 3, three clusters were categorized into the activity-nutrition SC, breath-sleep SC, and respiratory SC, based on the makeup of symptoms within each cluster.

**Table 3:** Symptom cluster analysis of patients with AECOPD at admission.

Symptom Cluster	Symptoms	Factor loading		
		Factor1	Factor2	Factor 3
**Activity-nutrition** SC	Decreased exercise tolerance	0.76		
	Limitation of activity	0.51		
	Fatigue	0.82		
	Loss of appetite	0.57		
	Weight loss	0.58		
**Breath-sleep** SC	Chest distress		0.59	
	Shortness of breath		0.76	
	Insomnia		0.71	
**Respiratory** SC	Cough			0.70
	Sputum			0.83
**Variance contribution rate(%)**		33.47	13.29	10.63
**Cumulative variance contribution rate(%)**		33.47	46.76	57.39

### 4.5 Correlation analysis of SCs with laboratory and imaging indicators

The activity-nutrition SC was correlated with white blood cell count and serum sodium and potassium levels (*P* < 0.05). White blood cell and eosinophil counts, serum potassium level, and pleural effusion were all associated with the breath-sleep SC (*P* < 0.05). The respiratory SC was associated with serum calcium and magnesium levels, the partial pressure of carbon dioxide, and C-reactive protein (CRP) level (*P* < 0.05). Detailed results are presented in Table 4.

**Table 4:** Correlation analysis of symptom clusters with laboratory and imaging indicators.

Variables	Activity-nutrition symptom cluster	Breath-sleep symptom cluster	Respiratory symptom cluster
**White blood cell count**	0.18*	0.19*	0.11
**Neutrophil**	0.11	−0.11	0.06
**Eosinophilcount**	0.09	0.19*	−0.04
**Serum sodium**	0.17*	0.00	0.02
**Serum potassium**	−0.23*	−0.16*	0.00
**Serum chlorine**	0.07	0.10	−0.04
**Serum calcium**	0.07	−0.05	0.20*
**Serum phosphorus**	0.09	−0.01	0.14
**Serum magnesium**	0.04	0.07	−0.19*
**PH**	0.15	−0.05	−0.07
**Partial pressure of oxygen**	−0.15	−0.03	0.03
**Partial pressure of carbon dioxide**	0.14	0.03	−0.20*
**Tumor markers**	0.09	0.10	0.08
**C-reactive protein**	0.12	0.05	0.30*
**Procalcitonin**	0.10	0.04	0.10
**Lung consolidation**	−0.01	0.05	−0.02
**Lymphadenopathy**	−0.04	0.14	0.16
**Pleural effusion**	0.14	0.16*	0.10
**Emphysema**	0.06	0.03	0.10
**Pulmonary nodule**	−0.15	−0.01	−0.01
**Coronary calcification**	0.02	−0.01	0.03
**Pulmonaryhypertension**	−0.00	0.02	−0.01

Note：* is *P*＜0.05.

### 4.6 Trajectory of SCs over time

Symptoms with prevalence ≥40 % on the third day after admission were included in the EFA, as shown in Supplementary Table 2. Three clusters were extracted, and the cumulative variance contribution rate was 56.32 %, whereas the individual variance contribution rates were 29.64 %, 14.26 %, and 12.42 %. Based on the composition of symptoms within the clusters, the three SCs were named as breath - sleep, activity - nutrition, and respiratory SCs, as indicated in Supplementary Table 3.

Seven symptoms with a prevalence of 40 % at discharge were included in the EFA. Upon extraction, two clusters exhibited variance contribution rates of 38.28 % and 15.02 % respectively, with a cumulative variance of 53.30 %. The two SCs were named as breath-activity and respiratory SC based on the makeup of the symptoms within the clusters, as shown in Table 5.

**Table 5:** Symptom clusters at discharge.

Symptom Cluster	Symptoms	Factor loading	
		Factor1	Factor2
**Breath - activity SC**	Chest distress	0.66	
	Shortness of breath	0.77	
	Decreased exercise tolerance	0.54	
	Limitation of activity	0.78	
	Fatigue	0.65	
**Respiratory SC**	Cough		0.80
	Sputum		0.78
**Variance contribution rate(%)**		38.28	15.02
**Cumulative variance contribution rate(%)**		38.28	53.30

Note:KMO = 0.73; Bartlett's test of sphericity was significant (*P* < 0.001).

### 4.7 Association of SCs with prognostic outcomes and disease burden

Correlation analysis results showed that the activity-nutrition SC was favourably linked with hospitalization cost (*r*_*s*_ = 0.23, *P* = 0.006). There was a positive correlation between the breath-sleep SC and hospitalization length (*r*_*s*_ = 0.22, *P* = 0.006) and cost (*r*_*s*_ = 0.29, *P* < 0.001), see Table 6 for details. However, no discernible variation was observed in the readmission risk among the three SCs.

**Table 6:** Association of SCs with prognostic outcomes and disease burden.

Symptom clusters	Hospitalization days		Hospitalization cost	
	*r*_*s*_	*P*	*r*_*s*_	*P*
**Activity - nutrition SC**	0.15	0.064	0.23	0.006
**Breath - sleep SC**	0.22	0.006	0.29	＜0.001
**Respiratory SC**	0.01	0.913	0.09	0.266

## 5 Discussion

### 5.1 Pattern of SCs in patients with AECOPD

Patients with AECOPD experienced three main SCs: activity-nutrition SC, breath-sleep SC, and respiratory SC. The activity - nutrition SC included fatigue, decreased exercise tolerance, limitation of activity, loss of appetite, and weight loss. Patients may experience weight loss and poor nutritional status due to hypoxia, inflammation, oxidative stress, inadequate intake, aging and continuous energy and protein consumption. Fatigue and motor dysfunction may ensue from this, along with muscle loss, respiratory muscle atrophy, and a deterioration in lung function. Conversely, inactivity may make it difficult to perform daily chores such as cooking and grocery shopping, which in turn increases the risk of malnutrition [28]. Simultaneously, we discovered a correlation between the severity of this SC and hospitalization costs, indicating the importance of screening for malnutrition and muscle loss in all patients with AECOPD [29,30].

Chest tightness, shortness of breath, and insomnia were components of the breath - sleep SC. Patients with AECOPD may experience chest tightness and shortness of breath from restricted bronchial airflow, imbalanced oxygen consumption, and decreased lung function. At the same time, reduced respiratory drive and chemoreceptor sensitivity during sleep can result in decreased tidal volume, increased upper airway resistance, and insufficient minute ventilation, leading to hypoxia, hypercapnia, increased respiratory effort, and ultimately contribute to respiratory disorders [31,32]. This in turn makes it difficult for them to fall asleep and stay asleep. We also discovered a positive correlation between the severity of this SC and the length and cost of hospitalization. This implies that co-existing symptoms will make AECOPD patients' disease burden greater, underscoring the urgency of implementing effective symptom cluster management.

The respiratory SC was composed of cough and sputum, consistent with the findings of Zhang et al. [33]. This condition is associated with airway inflammation and ciliary dysfunction [34,35]. Additionally, previous research has demonstrated that alterations in cough and sputum are indicators of COPD exacerbation and progression, significantly impacting patients' health outcomes, hospitalization rates, and mortality [36]. Therefore, it is necessary to reasonably evaluate SCs and manage them effectively to improve patients’ quality of life.

The composition of the SCs on admission and on the third day after admission remained largely consistent, based on the change trajectories of the SCs. However, some of the acute symptoms subsided or worsened as the subsequent treatment went on, which changed the number and makeup of SCs. So it is necessary to identify the key SCs in different disease stages to better prioritize symptoms and facilitate better assessment and management.

### 5.2 Underlying mechanism of SCs

Serum potassium and sodium levels, and white blood cell count were found to be associated with the activity-nutrition SC. Inflammation is the main cause of AECOPD. It releases inflammatory cells such as white blood cells, damages lung function, causes hypoxemia, and increases sympathetic nerve excitation compensation in the skeletal muscles, which makes patients less active and more fatigued [37]. In addition, patients with AECOPD often encounter a heightened incidence of malnutrition resulting from the elevated energy expenditure triggered by increased oxidative stress, hypoxia, and the inflammatory reactions [22]. Chronic malnutrition damages a patient's capacity for self-regulation and raises the possibility of electrolyte abnormalities [38]. As mediators of nerve and muscle signal transduction, abnormal sodium and potassium levels have an effect on signal transduction and can cause patients to get fatigued, lose their ability to perform, and require longer hospital stays [39]. Evidently, the symptoms in this cluster may have a similar physiological mechanism.

The breath-sleep SC was associated with eosinophil and white blood cell counts, serum potassium level, and pleural effusion. Our study's conclusions showed that 44.59 % of patients had abnormal eosinophil counts. The activation of eosinophilic granulocytes can release a variety of inflammatory factors, induce pulmonary eosinophilic inflammation, and damage airway epithelial cells. Several studies have shown that elevated eosinophil counts are associated with more severe dyspnea and an increased risk of exacerbations [40]. We also discovered a favorable correlation between the white blood cell count and duration of stay and the amount of eosinophils in patients with AECOPD, which is consistent with the finding of Chen et al. [41]. Patients with AECOPD also tend to have hypokalemia, which may be related to hypoxia and respiratory acidosis. This may cause respiratory muscle fatigue and aggravate the shortness of breath. Furthermore, we found that AECOPD patients with pleural effusion may experienced more severe chest tightness, shortness of breath, and insomnia. This correlation could be attributed to pleural effusion compressing lung tissue, which can lead to pulmonary impairment and limited thoracic compliance, thus compromising gas exchange [42,43].

The respiratory SC was related to CRP level, serum calcium and magnesium levels, and the partial pressure of carbon dioxide. As a common inflammatory marker, CRP levels are linked to inflammatory response activation and compromised immune function in patients with AECOPD [44]. When they are triggered, several inflammatory mediators can damage the respiratory mucosa and increase mucosal secretions. Reduced mucus clearance from ciliary dysfunction leads to mucus accumulation and possible bacterial colonization, which in turn triggers coughing and raises the frequency of attacks [45]. Increased airway secretions and clearance obstructions in AECOPD patients may also cause breathing dysfunction and elevated partial pressure of carbon dioxide [46]. Additionally, the increased inflammation can disturb the endocrine regulatory axis, leading to hypothyroidism and insufficient parathyroid hormone secretion. This disruption compromises the regulation of blood calcium levels, culminating in decreased calcium levels. These changes can further impact the release of inflammatory mediators, the generation of oxygen free radicals, the spasms of bronchial smooth muscle, and the exacerbation of cough and sputum production [47]. Serum magnesium level has also been shown to be associated with airway hyperresponsiveness and lung function impairment, both of which worsen respiratory symptoms in patients with AECOPD [48]. An in-depth exploration of the mechanisms underlying health issues is crucial for advancing the accuracy of nursing technology and decision-making.

### 5.3 Limitation

Our study also has the following limitations. First, our study was only conducted in a single tertiary hospital in Wenzhou, there was limited generalizability of the findings. Future multi-centre, large-sample investigations are required. Second, symptom assessment was primarily based on patient self-reports, which may have introduced a bias. Third, we did not consider the symptoms of patients who developed new complications during their hospitalization, which may have affected the study's results. Finally, the interpretation of the study's results may be affected by the fact that our longitudinal analysis of the trajectories of SC changes was limited to the hospitalization period and was unable to detect the trend of change that followed.

## 6 Conclusion

Patients with AECOPD were most affected by three SCs that influenced the length and cost of hospitalization: activity-nutrition SC, breath-sleep SC, and respiratory SC. Among these, activity-nutrition SC was associated with white blood cell count and serum sodium and potassium levels; the breath-sleep SC was related to white blood cell and eosinophil counts, serum potassium level, and pleural effusion; and the respiratory SC was related to serum calcium and magnesium levels, the partial pressure of carbon dioxide, and CRP level. These findings suggest that the symptoms within each cluster might share a common physiological mechanism. To improve patient prognosis and reduce financial burden, clinical nurses must be aware of AECOPD SCs and develop and implement evidence-based management strategies for SCs.

## 7 Relevance to clinical practice

Patients with AECOPD often present with severe symptoms, and such patients require additional clinical nursing care. The evaluation of co-existing symptoms should be part of the initial screening tool for patients with AECOPD. By understanding which symptoms commonly co-occur, nurses can focus on the nature of the relationship between a cluster of symptoms rather than the symptoms individually. Our study identified different SC patterns and trajectories in patients with AECOPD and explored the associations of these SCs with health outcomes and disease burden, which could help identify potential populations with poorer outcomes and optimize the allocation of healthcare resources. Finally, we explored the potential mechanisms of the SCs. Deeply exploring the occurrence mechanisms and intervention paths of health problems is of great significance in promoting the precision of nursing decision-making and technology.

## Funding statement

This study was supported by the 10.13039/501100012166National Key Research and Development Program of China grants 2016YFC1304000 (C Chen); The National Natural Scientific Foundation of China 82170017,82370085(C Chen); Zhejiang Provincial Key Research and Development Program 2020C03067 (C Chen).

## Data availability statement

Data can be made available on request with privatized health information.

## CRediT authorship contribution statement

**Mengying Xu:** Writing – original draft, Validation, Software, Project administration, Methodology, Investigation, Formal analysis, Data curation, Conceptualization. **Chunchun Yu:** Writing – original draft, Validation, Software, Methodology, Investigation. **Xiaona Lin:** Writing – original draft, Software, Methodology, Investigation. **Jiaqi Shi:** Writing – original draft, Supervision, Methodology, Investigation. **Xinyue Pang:** Writing – original draft, Validation, Software. **Zhi Chen:** Writing – original draft, Software, Methodology. **Hongjun Zhao:** Writing – review & editing, Validation, Software, Project administration, Methodology, Formal analysis, Data curation, Conceptualization. **Chengshui Chen:** Writing – review & editing, Visualization, Validation, Software, Resources, Project administration, Methodology, Funding acquisition, Formal analysis, Data curation, Conceptualization.

## Declaration of competing interest

The authors declare that they have no known competing financial interests or personal relationships that could have appeared to influence the work reported in this paper.
