# Supplementary material for: Identification of symptom clusters and change trajectories in patients with acute exacerbation of chronic obstructive pulmonary disease
Source: Heliyon. 2024 Jul 4;10(13):e33745. doi: 10.1016/j.heliyon.2024.e33745 (PMC11276911; doi:10.1016/j.heliyon.2024.e33745)
Supplement: Multimedia component 1 [file mmc1.docx]

**Supplementary Table**

Table 1 Prevalence and severity of abnormal laboratory and imaging indicators

| **Variables** | **Prevalence,n(%)** | **Severity (median, interquartile)** |
| --- | --- | --- |
| **Emphysema** | 126(85.14) | 4.00(4.00,4.00) |
| **Neutrophil** | 121(81.76) | 4.00(4.00,4.00) |
| **Tumor markers** | 111(75.00) | 1.00(0.75,1.00) |
| **Serum calcium** | 110(74.32) | 4.00(0.00,4.00) |
| **Partial pressure of carbon dioxide** | 93(62.84) | 4.00(0.00,4.00) |
| **Pulmonary hypertension** | 88(59.46) | 1.00(0.00,2.00) |
| **Coronary calcification** | 86(58.11) | 4.00(0.00,4.00) |
| **C-reactive protein** | 85(57.43) | 1.00(0.00,2.00) |
| **Partial pressure of oxygen** | 78(52.70) | 1.00(0.00,1.00) |
| **Eosinophilic count** | 66(44.59) | 0.00(0.00,4.00) |
| **Serum phosphorus** | 60(40.54) | 0.00(0.00,4.00) |
| **Leucocyte counts** | 52(35.14) | 0.00(0.00,4.00) |
| **Pleural effusion** | 44(29.73) | 0.00(0.00,1.00) |
| **PH** | 40(27.03) | 0.00(0.00,4.00) |
| **Serum potassium** | 37(25.00) | 0.00(0.00,0.25) |
| **Serum chlorine** | 32(21.62) | 0.00(0.00,0.00) |
| **Pulmonary nodule** | 30(20.27) | 0.00(0.00,0.00) |
| **Lymphadenopathy** | 24(16.22) | 0.00(0.00,0.00) |
| **Serum sodium** | 20(13.51) | 0.00(0.00,0.00) |
| **Serum magnesium** | 17(11.49) | 0.00(0.00,0.00) |
| **Lung consolidation** | 15(10.14) | 0.00(0.00,0.00) |
| **Procalcitonin** | 15(10.14) | 0.00(0.00,0.00) |

Table 2 Symptoms trajectory over time

| **Symptoms** | **Third day of admission** | | **At discharge** | | |
| --- | --- | --- | --- | --- | --- |
|  | **Prevalence,n(%)** | **Severity (median, interquartile)** | **Prevalence,n(%)** | | **Severity (median, interquartile)** |
| **Cough** | 109(90.08) | 1.00(1.00,1.00) | 95(64.19) | 1.00(0.00，1.00） | |
| **Sputum** | 108(89.26) | 1.00(1.00,4.00) | 108(72.97) | 1.00(0.00，1.00） | |
| **Chest distress** | 108(89.26) | 2.00(2.00,2.00) | 98(66.22) | 2.00(0.00，2.00） | |
| **Shortness of breath** | 118(97.52) | 2.00(2.00,2.00) | 137(92.57) | 2.00(2.00,2.00) | |
| **Cannot lie flat** | 40(33.06) | 0.00(0.00,4.00) | 15(10.14) | 0.00(0.00,0.00) | |
| **Fever** | 5(4.13) | 0.00(0.00,0.00) | 3(2.03) | 0.00(0.00,0.00) | |
| **Pain** | 11(9.09) | 0.00(0.00,0.00) | 4(2.70) | 0.00(0.00，0.00) | |
| **Decreased exercise tolerance** | 114(94.21) | 4.00(4.00,4.00) | 127(85.81) | 4.00(4.00，4.00) | |
| **Limited movement** | 119(98.35) | 2.00(2.00,4.00) | 139(93.92) | 2.00(2.00，2.00) | |
| **Hoarse** | 15(12.40) | 0.00(0.00,0.00) | 17(11.49) | 0.00(0.00,0.00) | |
| **Dysphagia** | 4(3.31) | 0.00(0.00,0.00) | 2(1.35) | 0.00(0.00,0.00) | |
| **Loss of appetite** | 79(65.29) | 1.00(0.00,1.00) | 33(22.30) | 0.00(0.00,0.00) | |
| **Weight loss** | 9(7.44) | 0.00(0.00,0.00) | 15(10.14) | 0.00(0.00,0.00) | |
| **Constipation** | 23(19.01) | 0.00(0.00,0.00) | 7(4.73) | 0.00(0.00,0.00) | |
| **Insomnia** | 59(48.76) | 0.00(0.00,4.00) | 23(15.54) | 0.00(0.00,0.00) | |
| **Drowsiness** | 7(5.79) | 0.00(0.00,0.00) | 3(2.03) | 0.00(0.00,0.00) | |
| **Fatigue** | 96(79.34) | 4.00(4.00,4.00) | 64(43.24) | 0.00(0.00,4.00) | |
| **Dysphoria** | 12(9.92) | 0.00(0.00,0.00) | 4(2.70) | 0.00(0.00,0.00) | |
| **Anxiety** | 33(27.27) | 0.00(0.00,4.00) | 16(10.81) | 0.00(0.00,0.00) | |
| **Limb swelling** | 17(14.05) | 0.00(0.00,0.00) | 14(9.46) | 0.00(0.00,0.00) | |

Table 3 Symptom clusters on the third day of admission

| **Symptom Cluster** | **Symptoms** | **Factor loading** | | |
| --- | --- | --- | --- | --- |
|  |  | **Factor1** | **Factor2** | **Factor3** |
| **Activity-nutrition SC** | Decreased exercise tolerance | 0.71 |  |  |
|  | Fatigue | 0.79 |  |  |
|  | Loss of appetite | 0.54 |  |  |
| **Breath-sleep SC** | Chest distress |  | 0.65 |  |
|  | Shortness of breath |  | 0.70 |  |
|  | Insomnia |  | 0.74 |  |
|  | Limitation of activity |  | 0.69 |  |
| **Respiratory SC** | Cough |  |  | 0.71 |
|  | Sputum |  |  | 0.73 |
| **Variance contribution rate(%)** | | 29.64 | 14.26 | 12.42 |
| **Cumulative variance contribution rate(%)** | | 29.64 | 43.90 | 56.32 |

Note: KMO=0.72；Bartlett’s test of sphericity was significant (*P* < 0.001)
